# Supplementary material for: Stacking Model‐Based Classifiers for Dealing With Multiple Sets of Noisy Labels
Source: Biom J. 2025 Mar 12;67(2):e70042. doi: 10.1002/bimj.70042 (PMC11898607; doi:10.1002/bimj.70042)
Supplement: Supplementary file 1 — Supporting Information [file BIMJ-67-e70042-s001.zip › density-based-ensemble-model-main/competitors_real_data/netlab-master/netlab-master/help/demhmc3.htm]

Netlab Reference Manual demhmc3


# demhmc3

## Purpose

Demonstrate Bayesian regression with Hybrid Monte Carlo sampling.

## Synopsis

```
demhmc3
```

## Description

The problem consists of one input variable `x` and one target variable
`t` with data generated by sampling `x` at equal intervals and then
generating target data by computing `sin(2*pi*x)` and adding Gaussian
noise. The model is a 2-layer network with linear outputs, and the hybrid Monte
Carlo algorithm (with persistence) is used to sample from the posterior
distribution of the weights. The graph shows the underlying function,
300 samples from the function given by the posterior distribution of the
weights, and the average prediction (weighted by the posterior probabilities).

## See Also

`demhmc2`, `hmc`, `mlp`, `mlperr`, `mlpgrad`

---

**Pages:**
Index


---

Copyright (c) Ian T Nabney (1996-9)
